# Different Bone Healing Effects of Undifferentiated and Osteogenic Differentiated Mesenchymal Stromal Cell Sheets in Canine Radial Fracture Model

**DOI:** 10.1007/s13770-017-0092-8

**Published:** 2017-11-15

**Authors:** Yongseok Yoon, Imdad Ullah Khan, Kyeong Uk Choi, Taeseong Jung, Kwangrae Jo, Su-Hyung Lee, Wan Hee Kim, Dae-Yong Kim, Oh-Kyeong Kweon

**Affiliations:** 1 Department of Veterinary Surgery, College of Veterinary Medicine, Seoul National University, 1 Gwanak-ro, Gwanak-gu, Seoul, 08826 Korea; 2 Department of Veterinary Pathology, College of Veterinary Medicine, Seoul National University, 1 Gwanak-ro, Gwanak-gu, Seoul, 08826 Korea; 3 BK21 PLUS Program for Creative Veterinary Science Research, Research Institute for Veterinary Science and College of Veterinary Medicine, Seoul National University, 1 Gwanak-ro, Gwanakgu, Seoul, 08826 Korea

**Keywords:** MSCs sheet, Undifferentiation, Osteogenic differentiation, Bone healing, Dog

## Abstract

Cell sheets technology is being available for fracture healing. This study was performed to clarify bone healing mechanism of undifferentiated (UCS) and osteogenic (OCS) differentiated mesenchymal stromal cell (MSC) sheets in the fracture model of dogs. UCS and OCS were harvested at 10 days of culture. Transverse fractures at the radius of six beagle dogs were assigned into three groups (n = 4 in each group) i.e. UCS, OCS and control. The fractures were fixed with a 2.7 mm locking plate and six screws. Cell sheets were wrapped around the fracture site. Bones were harvested 8 weeks after operation, then scanned by micro-computed tomography (micro-CT) and analyzed histopathologically. The micro-CT revealed different aspects of bone regeneration among the groups. The percentages of external callus volume out of total bone volume in control, UCS, and OCS groups were 42.1, 13.0 and 4.9% (*p* < 0.05) respectively. However, the percentages of limbs having connectivity of gaps were 25, 12.5 and 75% respectively. In histopathological assessments, OCS group showed well organized and mature woven bone with peripheral cartilage at the fracture site, whereas control group showed cartilage formation without bone maturation or ossification at the fracture site. Meanwhile, fracture site was only filled with fibrous connective tissue without endochondral ossification and bone formation in UCS group. It was suggested that the MSC sheets reduced the quantity of external callus, and OCS induced the primary bone healing.

## Introduction

Mesenchymal stromal cells (MSCs) have potentials for bone regeneration [1–3]. Previous reports indicated that MSCs could repair damaged tissue by replacing damaged cells [3]. Furthermore, MSCs may indirectly contribute to tissue repair through the regulation of inflammation and secretion of growth factors [4, 5]. Injection of suspended MSCs for delivery to the target tissues has the disadvantage of MSCs disappearing from the system, soon after transplantation [6]. Cell sheets technology using temperature-responsive culture dishes [7, 8] or using ascorbic acid 2-phosphate (A2-P) has been proposed, and the stemness and transdifferentiation of cell sheets has been proven [9–11].

Cell sheets retain cell-to-cell junctions and are able to secrete abundant endogenous extracellular matrix (ECM) [9, 10] and form multiple layers [12]. An intact cell sheet structure guarantees homeostasis of the cellular microenvironment during delivery of cytokines over a period of time to accelerate tissue repair [6, 13]. Cell sheets have been evaluated in the regeneration of the ligament, cornea, urinary bladder, liver, heart and bone [14].

Osteogenic differentiated cell sheets (OCSs) have been used for bone regeneration, and to promote bone healing [15–20]. OCSs cultured over 10 days can easily be folded away because of cellular aggregation, but undifferentiated cell sheets (UCSs) could be cultured for longer [13, 21]. Our previous study showed that OCSs could produce more osteogenic-related factors than UCSs at 7–10 days after differentiation and this time was more suitable for application at the fracture site [21].

Pins have been used as an internal fixation material in studies for evaluation of effect of cell sheets on bone regeneration because more rigid fixation such as plates and screws could not be applied in experimental animals such as mice, rats and rabbits [15, 17–19, 22–24]. The bone healing pathway by unstable fixation like pins is expected to be secondary bone healing due to cartilaginous callus formation however there is no primary bone healing through intramembranous ossification [25].

It may be suggested that the type of cell sheets and fixation method affect the bone healing pathway. This study was performed to clarify whether UCSs and OCSs under rigid fixation promote bone healing by different pathways.

## Materials and methods

### Isolation and culture of canine adipose tissue derived mesenchymal stromal cells (ad-MSCs)

All animal experimental procedures were approved by the Institutional Animal Care and Use Committee of Seoul National University (SNU-150423-6) and (SNU-161108-5). Canine ad-MSCs were obtained according to the methods described in our previous paper [26]. In brief, fat tissues were harvested aseptically from the gluteal subcutaneous fat of beagle dogs. The tissues were washed with Dulbecco’s phosphate-buffered saline (DPBS, Gibco, Grand Island, NY, USA), and immersed in collagenase type I (1 mg/ml; Sigma-Aldrich, St Louis, MO, USA) for 2 h at 37 °C. After treatment with collagenase type 1, the samples were washed with phosphate-buffered saline (PBS) and centrifuged at 300×*g* for 10 min. The pellet was resuspended and it was filtered through a 100-µm nylon mesh. Those cells were seeded on the dishes and incubated in low-glucose Dulbeco’s modified Eagle’s medium (DMEM, HyClone, Logan, UT, USA) with 10% fetal bovine serum (FBS, Gibco BRL, Grand Island, NY, USA) and 1% penicillin/streptomycin (P/S, Hyclone) at 37 °C with 5% humidified CO_2_. The medium was changed every 2 days until the dishes became confluent with cells. After cells reached 90% confluence, they were subcultured.

### Preparation of cell sheets

The cells at passage 3 were seeded on 100 mm dishes and cultured in low DMEM with FBS and P/S. The medium was changed to the differentiation medium when the confluency reached 90% which contains 1 × 10^6^ cells. To prepare UCSs, the differentiation medium consisted of low DMEM, 10% FBS, 1% P/S and 50 µg/ml A2-P (Sigma-Aldrich). In the case of OCSs, the differentiation medium consisted of high DMEM, 10% FBS, 1% P/S, A2-P 50 µg/ml and 10^−7^M dexamethasone (Dex, Sigma-Aldrich). The medium was changed every 2 days and at 10 days from differentiation, the cell sheets were washed twice with dPBS and harvested. These harvested cell sheets were applied at the fracture site.

### Real-time polymerase chain reaction (real-time PCR)

To compare the factors related to inflammation and osteogenicity at the mRNA level, undifferentiated MSCs (u-MSCs) were cultured as a control. In the case of u-MSCs, total RNA was extracted when the cells reached 90% confluency. In the case of UCSs and OCSs, total RNA was extracted when they reached 10 days of culture. Total RNA was isolated using a Hybrid-R RNA Extraction Kit (GeneAll, Seoul, Republic of Korea) and the RNA concentration were determined by measuring the light absorbance at 260 nm using ImplenNanoPhotometer (model 1443, Implen GmbH, Munich, Germany). One milligram of total RNA was used to synthesize cDNA with PrimeScript II First-strand cDNA Synthesis kit (Takara, Otsu, Japan). The cDNA obtained was then amplified via real-time PCR using an ABI StepOnePlus Real-time PCR System (Applied Biosystems) and SYBR Premix EX Taq (Takara, Otsu, Japan). The primers used for real-time PCR are listed in Table 1, and GAPDH was used as the housekeeping gene. Genes related to inflammation, including interlukin-6 (IL-6), interlukin-10 (IL-10), cyclooxygenase-2 (COX-2), tumor necrosis factor-α (TNF-α) and hepatocyte growth factor (HGF), and factors related to osteogenicity, including runt-related transcription factor 2 (RUNX2), bone morphogenetic protein 7 (BMP7), and transforming growth factor beta (TGF-β) were assessed by real-time PCR.

**Table 1:** Canine primers used for real-time PCR

Primer	Forward	Reverse
GAPDH	CATTGCCCTCAATGACCACT	TCCTTGGAGGCCATGTAGAC
BMP-7	TCGTGGAGCATGACAAAGAG	GCTCCCGAATGTAGTCCTTG
RUNX2	TGTCATGGCGGGTAACGAT	TCCGGCCCACAAATCTCA
TGF-B	CTCAGTGCCCACTGTTCCTG	TCCGTGGAGCTGAAGCAGTA
IL-10	CCTGGGAGAGAAGCTCAAGA	TGTTCTCCAGCACGTTTCAG
1L-6	TTTTCTGCCAGTGCCTCTTT	GGCTACTGCTTTCCCTACCC
Cox-2	ACCCGCCATTATCCTAATCC	TCGGAGTTCTCCTGGCTTTA

### Induction of fracture and application of cell sheets

Eight male beagle dogs (age, 2–3 years, body weight 8.0 ± 0.5 kg) were used in the study. Animals were pre-medicated with 30 mg/kg cefazolin (Chong Kun Dang Pharmaceutical Co., Seoul Korea), 4 mg/kg tramadol (Samsung Pharmaceutical Co., Seoul, Korea) and 0.5 mg/kg famotidine (Dong-A ST, Seoul, Korea). They were pre-anesthetized with 3 mg/kg alfaxalone (Jurox Pty. Limited Co., Rutherford, Austalia) and intubated with an endotracheal tube and anesthesia was maintained with isoflurane (Choongwae Pharmaceutical Co., Seoul, Korea).

**Fig. 1:**
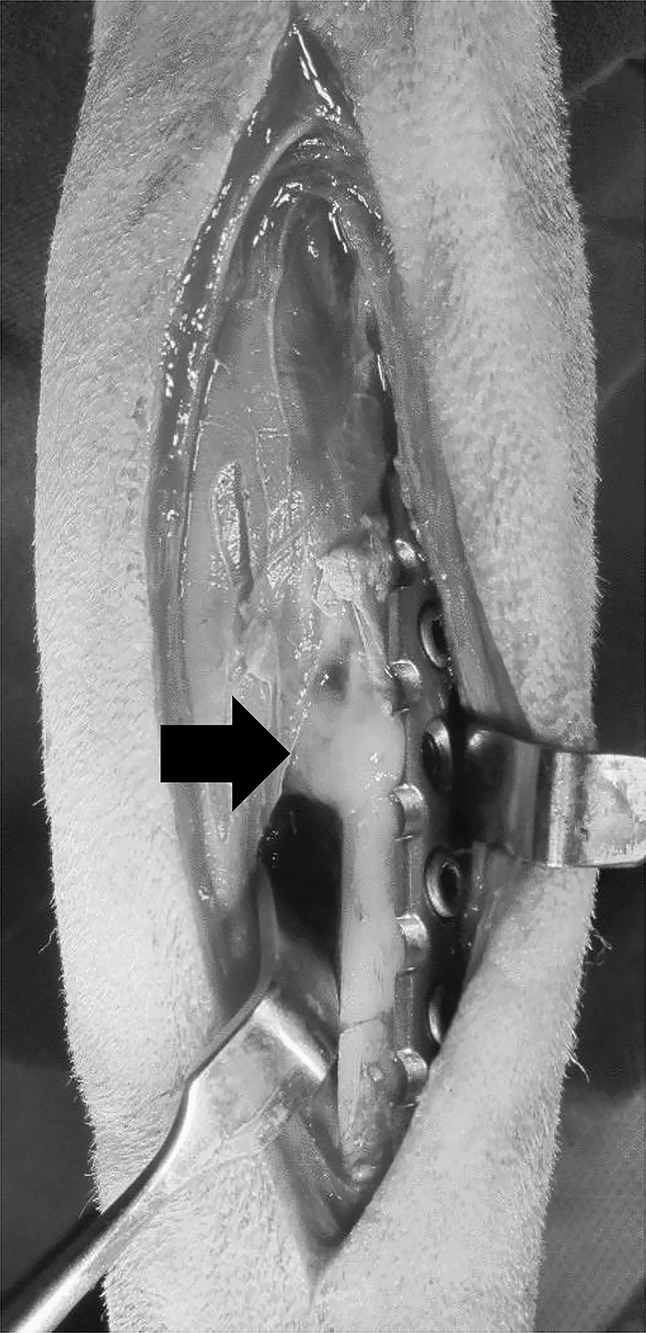
Implantation of cell sheets. Radii were fixed with seven hole locking plate. In UCS and OCS groups, cell sheets were applied to fill and surround the gap. Black arrow indicates cell sheets

Transverse fractures were induced at the middle of both radii with an oscillating saw. Those fracture sites were fixed with seven holes, a 2.7 mm locking plate (LP), and six locking screws. (BS.COREM Co., Korea) The limbs of six dogs were randomly divided into three groups (n = 4 for each group): UCS, OCS and no treatment (control). Three layers of cell sheets were applied to fill and surround the gap (Fig. 1). Dogs were subjected to post-operative care for 7 days. The skin incision was dressed every day until recovery and 8 weeks after surgery the limbs were harvested. Additional two dogs were randomly divided to control, UCS and OCS groups for evaluation of early bone healing. Limbs of two dogs were harvested at 4 weeks after surgery.

### Radiographic examination and micro-computed tomography (micro-CT)

The radiographic examination was conducted before the operation and every week after the operation for 8 weeks. Then the dogs were euthanized and their bones were harvested. These samples were fixed in 10% neutral buffered formalin (Sigma-Aldrich, St Louis, MO, USA) and were scanned after careful dissection and removal of the plates using a micro-CT system (SKYSCAN 1172: HIGH RESOLUTION DESK-TOP MICRO-CT). Briefly, the samples were scanned using a protocol that utilizes high resolution X-ray energy settings of 85KVP and 118 µA with a pixel size of 31.8 µm. Bone was considered at threshold 60–255. Quantification of bone volume and external callus was performed by the CTan software (Brucker micro-CT, version 1.14.4.1). Using 200 layers, measurements were taken of the volume 3.5 mm above and below the fracture.

### Histopathological and histomorphometry analysis

Segments of the bone, which were harvested at 4 weeks after surgery, including fracture sites were processed for histological analysis without decalcification. Samples were fixed in 10% neutral buffered formalin for 2 weeks and processed for resin embedding using an Technovit 7200 resin (Heaeus KULZER, Germany) and hardening with UV embedding system (KULZER EXAKT 520, Germany). Longitudinal sections were cut into sagittal planes using EXAKT diamond cutting system (EXAKT 300 CP, Germany). The central longitudinal sections from each radius were ground to a thickness of 40 µm and stained with hematoxylin and eosin (H&E).

Harvested bones, which were harvested at 8 weeks after surgery, were decalcified in 8% nitric acid, embedded in paraffin and sectioned at 4 µm for hematoxylin and eosin staining or 8 µm for Masson’s trichrome staining. Samples were evaluated for the degree of formation of cartilage, bone and fibrotic tissues at the fracture site. The slide images were analyzed using ImageJ (version 1.37, National Institutes of Health, Bethesda, MD, USA).

### Statistical analysis

Data is presented as mean ± standard deviation (SD). The IBM SPSS Statistics 23 statistical software was used to analyze the data (SPSS INC., Chicago, IL, USA). The difference between groups was analyzed using the Kruskal–Wallis test. The Mann–Whitney U test was used to confirm the differences between groups. Statistically significant values were defined as *p* < 0.05.

## Results

### Expressions of osteogenic and inflammatory factors

**Fig. 2:**
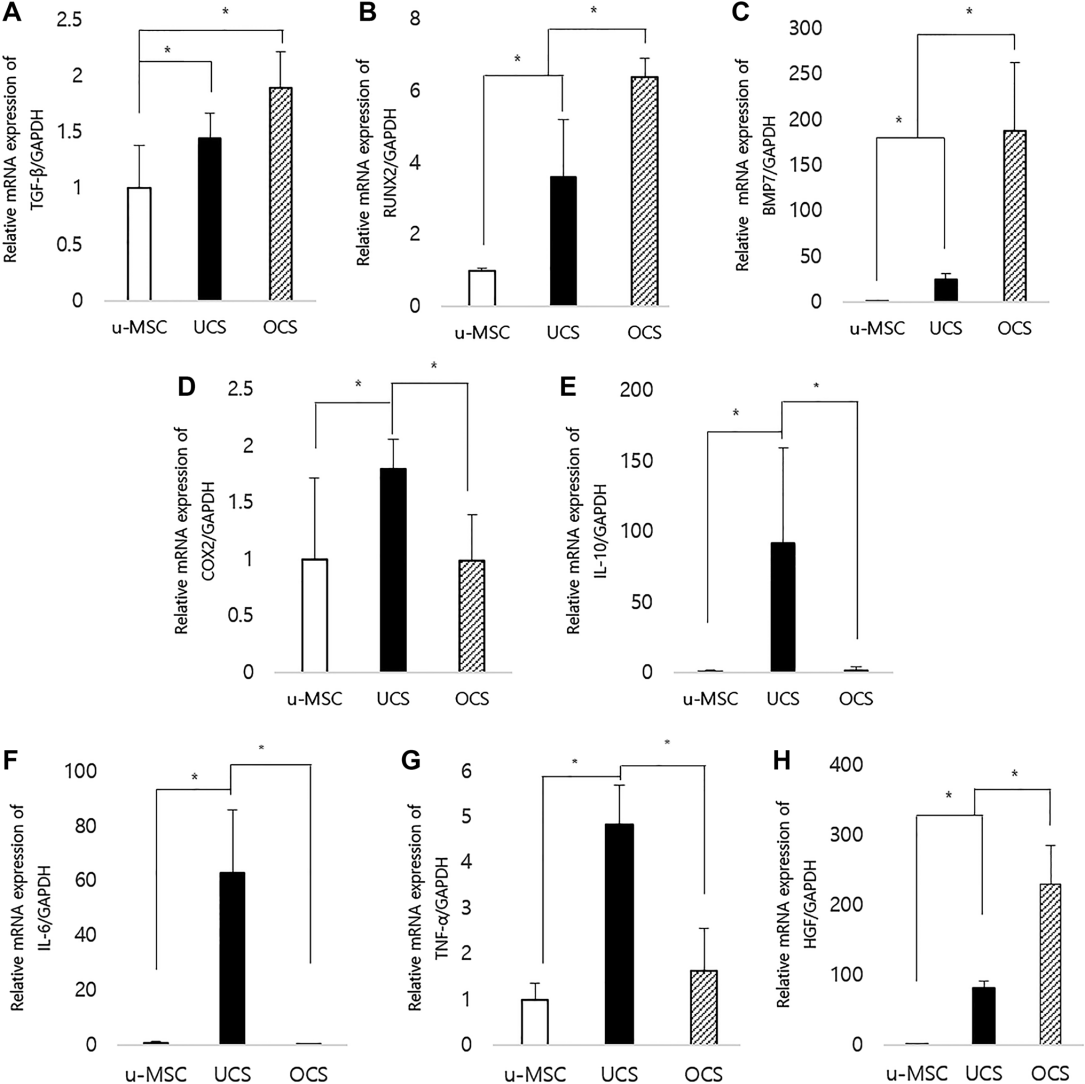
Osteogenic differentiation and inflammatory-related gene expression profiles of u-MSC, UCS and OCS at 10 days of culture. **A** The expressions of TGF-β mRNA in UCS and OCS groups were considerably upregulated compared to the u-MSC. **B**, **C** RUNX2 and BMP7 were up-regulated in OCS compared to both u-MSC and UCS. **D**–**G** Expressions of COX-2, IL-6, IL-10 and TNF-α in UCS were markedly upregulated compared to u-MSC and OCS. **H** In case of HGF, both group showed upregulated compare to control. Each bar represents the mean ± SD. ‘*’ represents statistically significant difference (*p* < 0.05)

The TGF-β mRNA expression level in UCS and OCS was significantly upregulated, compared to u-MSCs (*p* < 0.05, Fig. 2A). RUNX2 and BMP7 expressions in OCS were up-regulated compared to both u-MSCs and UCS (*p* < 0.05, Fig. 2B, C). In UCS, expression levels of COX-2, TNF-α, IL-6 and IL-10 were markedly upregulated compared to those in u-MSCs and OCS (*p* < 0.05, Fig. 2D–G). HGF expression level in OCS was increased significantly than those in UCS and u-MSCs (*p* < 0.05, Fig. 2H). UCS also had higher expression of HGF than u-MSCs.

### Changes in callus formation at fracture sites

**Fig. 3:**
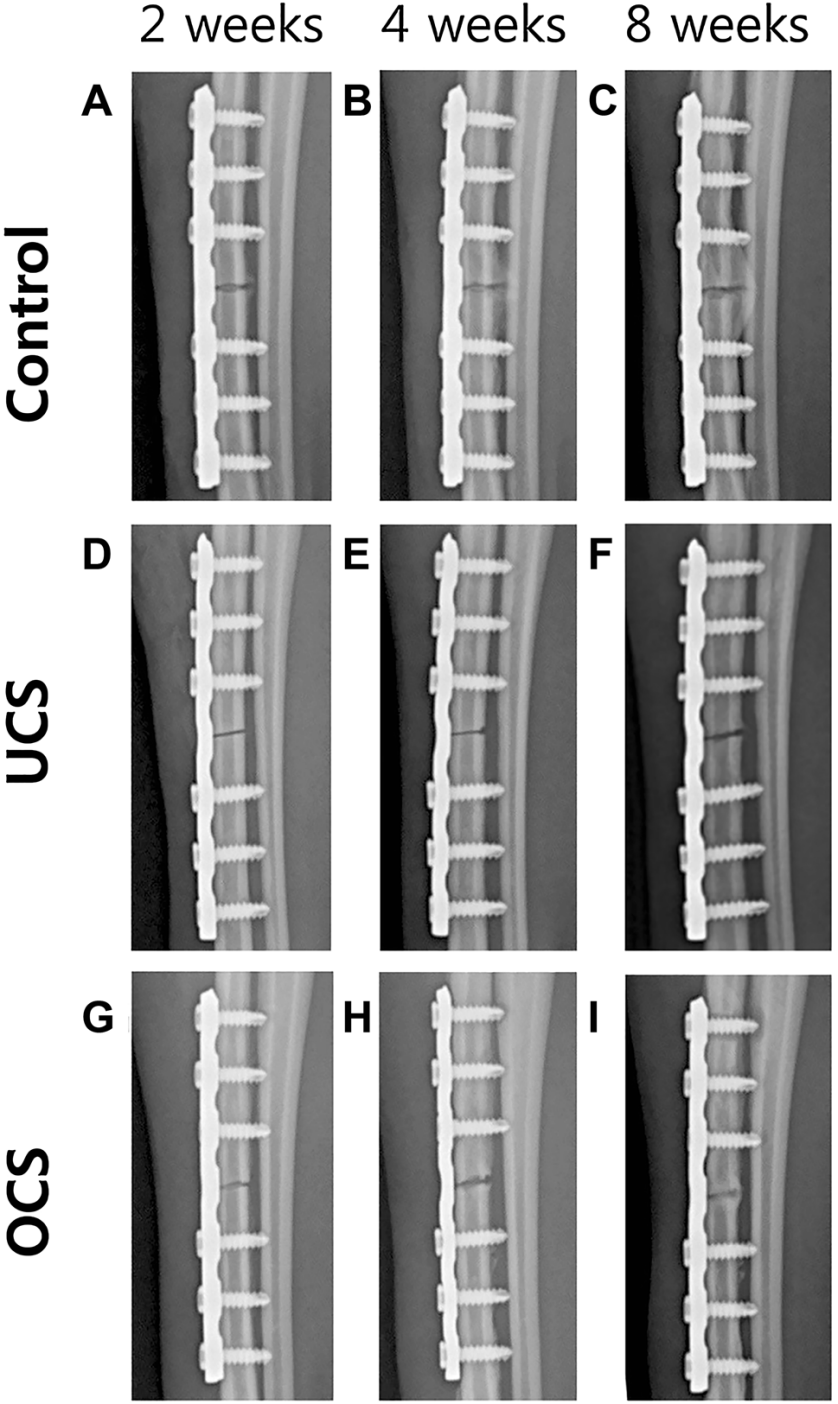
Radiographic changes of bone healing in relation to weeks. **A**,** D**,** G** Control group at 2 weeks showed external callus but other groups did not. **B**, **E** External callus in control group became larger but osteolysis was observed in UCS group. **C** The callus at 8 weeks was larger than at 4 weeks in control group. **F**, **I** But in UCS and OCS groups, external callus was not observed as much as control group and OCS group at 8 weeks showed connectivity between the gap

X-ray results showed that the implants were in proper position for the entire course of the experimental study period, i.e., 8 weeks. All the groups showed a healing response. At 2 weeks, the control group showed an external callus, while at 8 weeks the callus size definitely increased (Fig. 3A–C). In contrast, the UCS and OCS groups did not show abundant callus formation as seen in control group (Fig. 3D–I).

**Fig. 4:**
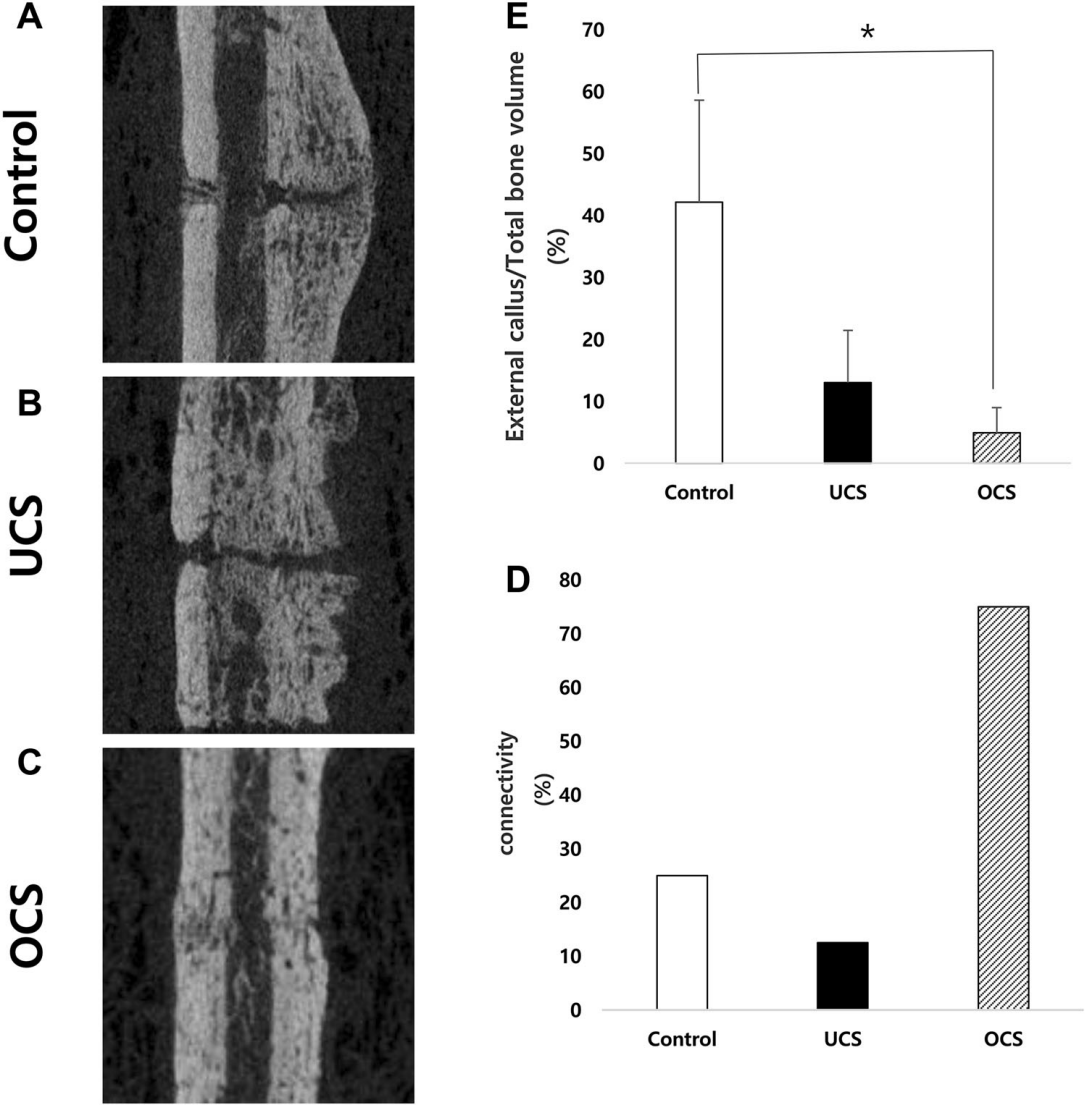
Micro-CT images at fracture sites and morphologic analysis of bone healing. Large bony callus formation was observed in the control but connectivity between cortical bones was not. **A**–**C** UCS and OCS groups showed no significant callus formation. In OCS group, there was connectivity between cortices, and the gaps were filled with materials which were similar attenuation as normal bone.** D** The percentage of external callus volume out of total bone volume in OCS group significantly decreased compared to the control (*p* < 0.05).** E** The percentages of limbs which had connectivity between cortices were 25, 12.5 and 75% respectively

Micro-CT analysis showed a large bony callus in control group (Fig. 4A). Compared to the control, the callus size was less in UCS and OCS groups (Fig. 4B, C). The percentages of external callus volume out of total bone volume were 42.1, 13.0 and 4.9% in the control, UCS and OCS groups, respectively. Callus size in OCS group was significantly less than in the control group (*p* < 0.05, Fig. 4D). The percentages of gap connectivity of cortices in each group i.e. 25% (2/8) in control, 12.5% (1/8) in UCS and 75% (6/8) in OCS groups (Fig. 4E).

### Histopathological assessment

**Fig. 5:**
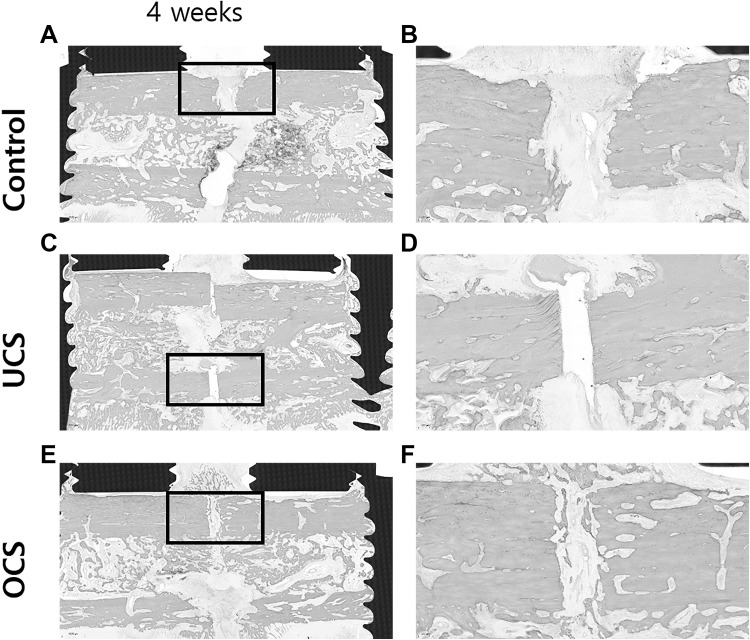
H&E staining of fracture sites at 4 weeks without decalcification.** E**,** F** The OCS treated group showed peripheral cartilage at the fracture site at 4 weeks. **A**–**D** However, control and UCS-treated groups did not have bone healing sign at the fracture site

At 4 weeks after bone injury, the OCS-treated group showed peripheral cartilage at the fracture site (Fig. 5E, F). However, control and UCS-treated groups did not represent healing signs at the fracture site (Fig. 5A–D).

**Fig. 6:**
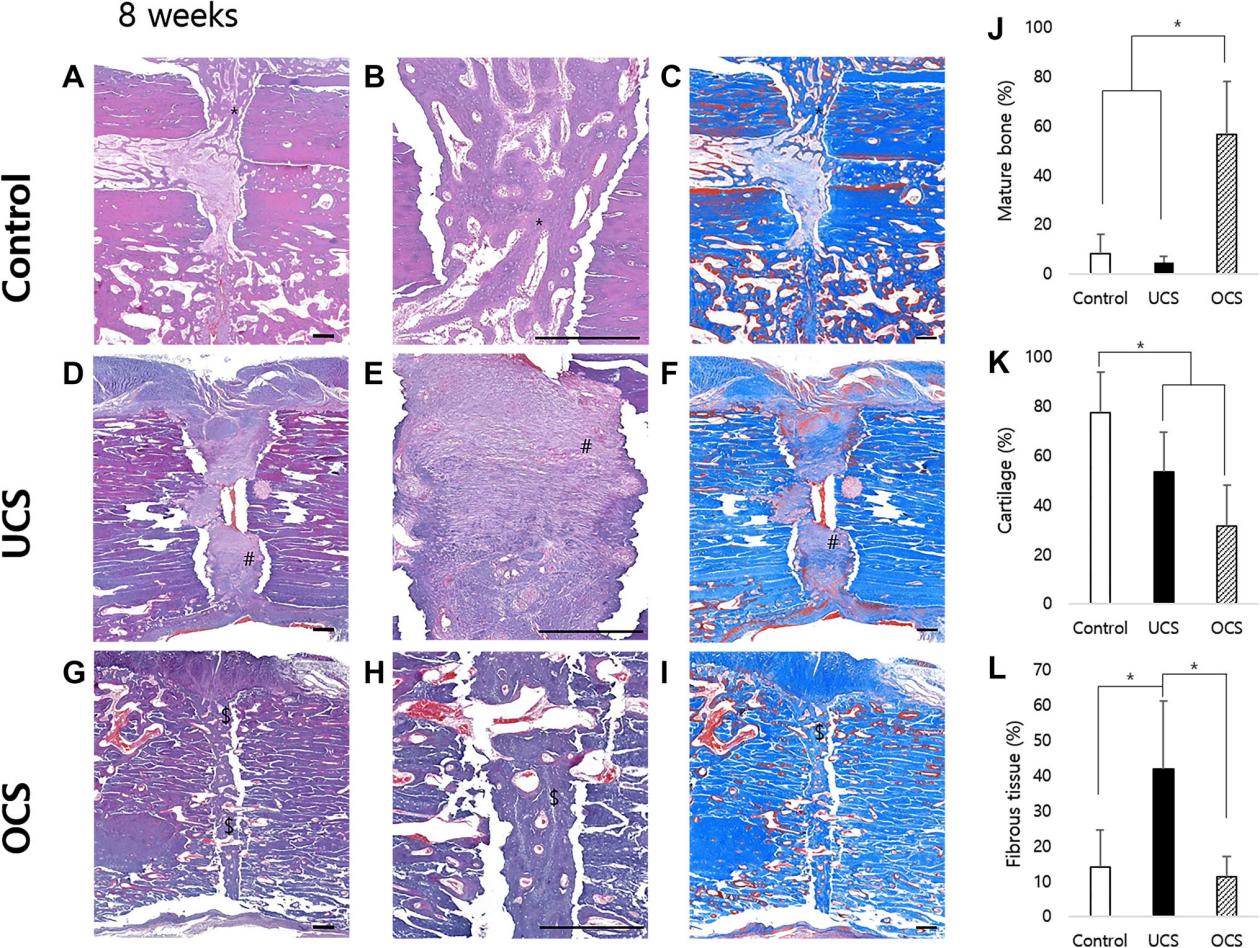
Histopathological findings and histomorphometric analysis of fracture sites at 8 weeks post cell sheets grafting. **A**–**C** Control group showed cartilage formation (*) without ossification at fracture site. **D**–**F** UCS group showed that the fracture site was filled with fibrous connective tissue (#) with less endochondral ossification or bone formation. **G**–**I** OCS group showed that the fracture site was well organized with peripheral cartilage and mature woven bone. **J** Scale bar: 100 μm. OCS group showed significant increased mature bone compare to control and UCS group. **K** Control group showed definitely increased cartilage compare to OCS. **L** Fibrous connective tissue was increased in UCS group. Each bar represents the mean ± SD. * represent a statistically significant difference (*p* < 0.05). H&E staining, Masson’s trichrome staining

At 8 weeks after bone injury, the OCS-treated group showed well organized and mature woven bone with peripheral cartilage at the fracture sites (Fig. 6G–I). The control group showed early phase of bone healing, that was the formation of cartilage with little bone maturation or ossification at the fracture site (Fig. 6A–C). In the UCS-treated group, the fracture site was only filled with fibrous connective tissues without endochondral ossification and bone formation (Fig. 6D–F).

Histomorphometric analysis of fracture sites showed that the percentage of mature bone in the OCS group (Fig. 6J, *p* < 0.05), percentage of cartilage in the control group (Fig. 6K, *p* < 0.05) and percentage of fibrous connective tissue in the UCS group (Fig. 6L, *p* < 0.05) were significantly higher than those in each of the other groups.

## Discussion

We tried to clarify the mechanism of fracture healing with cell sheets. It was found that cell sheet types and fixation methods have different ways of bone regeneration. In the present study, although fracture sites were fixed with the same fixation system (locking plate and screws) bone healing responses were different between the groups. At 8 weeks after surgery, cartilage, fibrous connective tissues and bone were found at fracture sites in control, UCS and OCS groups, respectively.

In previous reports, osteogenic cell sheets with pin fixation were found to promote bone healing by large callus formation at the fracture site [15, 19]. In the present study, abundant external callus was found in the control group compared to UCS and OCS groups, although the fixation was more rigid with bone plates and screws unlike pins used in previous studies. The control group showed secondary bone healing that induced external callus formation as seen on X-ray and micro-CT. Abundant callus formation in the control is probably due to periosteal reaction after surgical injury and the presence of micromotion at the fracture site. Bone has periosteum in the surface which has three layers; osteoblast, cambium and fibrous layers from the outer cortex [27]. Preexisted osteoblasts and osteogenic progenitor cells from vessels of cambium participate in the formation of callus. Callus formation is also proportional to the instability of the fracture [28].

Gap healing, a type of primary bone healing characterized by formation of bone at the fracture gap following little secondary bone healing, occurs when the fixation is rigid and the interfragmentary gap is approximately 1 mm or less [25]. If considerable strain is applied, the micromotion may occur at the fracture site, even though the gap is less than 1 mm which may lead to an abundant callus formation. The OCS group showed gap healing with the formation of bone and less external callus despite the same fixation as in the control group. Increase in the expression level of BMP 7 in OCSs indicated that the cells might be mature osteoblasts [21, 29]. Furthermore, BMP 7 factor secreted by mature osteoblasts could stimulate mobilization of osteogenic progenitor cells near the fracture site [30, 31]. The mature osteoblasts could produce bone in the intercortical gap [18], which would confer stability at the fracture site. OCSs have immune modulatory factors as well as osteogenic factors [32]. Less abundant callus formation in OCS group than the control suggested that immune modulatory factors might reduce the periosteal reaction to bone injury. The TGF-β mRNA expression levels in OCS group were significantly upregulated compared to u-MSCs. Although TGF-β is a pleiotropic cytokine with potent regulatory and inflammatory activity [33] it might function as an anti-inflammatory cytokine for OCS derived bone healing. Moreover, HGF expression level in OCS group was increased. It was reported that HGF promoted osteogenic differentiation and was a necessary component for the establishment of osteoblast mineralization [34]. Therefore, in the present study, the gap was filled with bone with least external callus formation.

UCS group showed lesser callus formation and more connective fibrous tissues than the control group. UCS showed significantly higher level of inflammatory cytokines including COX-2, TNF-α, and IL-6 which might negatively affect bone formation. It has been reported that inflammatory cytokines including IL-6 and TNF-α activate osteoclast and osteoclastogenesis [35–40]. It has also been reported that proinflammatory cytokines including TNF-α, inhibit osteogenic differentiation from stem cells [41]. In the present study the delayed bone healing with less callus formation might be due to the abundant expression of proinflammatory cytokines by UCS. It was reported that COX-2 and IL-6 expressions were significantly higher in freeze–thawed cells than fresh MSCs and that these increases might be due to heat stress during the thawing process [43]. Expression of IL 10 in UCS group was elevated than other groups. IL 10 has immune modulatory effects [42]. Immunomodulatory effect of IL-10 might have relation of causing the delayed bone healing. However, further studies on the effect of increase in proinflammatory and immunomodulatory cytokines in UCSs on bone healing are needed.

On the basis of these results, the difference in the osteogenic ability of OCSs and other groups might be due to early bone formation between the fracture gaps which stabilize the fracture site and result in less callus formation. OCSs and UCSs have different *in vivo* osteogenic abilities in fracture repair; that is, direct healing for OCSs and delayed healing for UCSs. Therefore, we suggest OCS is therapeutically more desirable for fracture healing.
